# Single-Dose L-Lysine-Induced Pancreatitis Followed by Pancreatic Neoplastic Progression in Adult *Kras/Trp53*-Driven Mice

**DOI:** 10.3390/mps9040116

**Published:** 2026-08-10

**Authors:** Akihiro Kaneda, Masahiro Yoshida, Yoshiya Kawaguchi, Kenichiro Furuyama

**Affiliations:** Center for iPS Cell Research and Application, Kyoto University, Kyoto 606-8507, Japan

**Keywords:** pancreatic ductal adenocarcinoma, protocol, pancreatitis, L-lysine, adult-onset mouse model

## Abstract

Pancreatic ductal adenocarcinoma (PDAC) remains a highly lethal malignancy, highlighting the need for reproducible experimental systems that model pancreatic injury and subsequent neoplastic progression. Adult-onset genetically engineered models in which oncogenic *Kras* and mutant *Trp53* are induced in the pancreas provide a relevant platform for studying PDAC pathogenesis, but efficient neoplastic progression often requires concomitant pancreatic injury. Cerulein-induced pancreatitis is widely used to provide an injury-associated inflammatory stimulus that promotes PDAC progression; however, repeated-injection regimens are labor-intensive and increase cumulative handling stress in animals. Here, we describe a dose-optimized, single-dose L-Lysine protocol designed to induce acute pancreatitis and characterize subsequent pancreatic neoplastic progression in adult-onset *Kras/Trp53*-driven mice. Tamoxifen-treated *Ptf1a*^CreER/+^; *Kras*^LSL-G12D/+^; *Trp53*^LSL-R172H/+^; Rosa26-RFP mice received a single intraperitoneal injection of L-Lysine, followed by biochemical, histological, and immunohistochemical assessment of pancreatic injury and tumor development. A single 2.0 g/kg L-Lysine injection induced sublethal acute pancreatitis characterized by increased serum pancreatic enzymes, interstitial edema, inflammatory cell infiltration, acinar cell necrosis, and rapid mitochondrial alterations. During 3–6 months of follow-up, mice developed multifocal acinar-to-ductal metaplasia, PanIN lesions, invasive PDAC, and peritoneal dissemination. This protocol provides a simple, synchronized, and less labor-intensive model for studying pancreatic injury and subsequent neoplastic progression in adult-onset *Kras/Trp53*-driven mice.

## 1. Introduction

Pancreatic ductal adenocarcinoma (PDAC) remains one of the most lethal malignancies, and experimental models that recapitulate its stepwise development are essential for understanding tumor initiation, progression, invasion, and metastasis. PDAC is generally considered to arise through a multistep process involving preinvasive epithelial lesions, particularly pancreatic intraepithelial neoplasia (PanIN). PanIN lesions are classified according to increasing degrees of architectural and cytological atypia, ranging from low-grade PanIN-1A and PanIN-1B lesions to PanIN-2 and carcinoma-in situ-like PanIN-3 lesions [1]. Molecular analyses of human pancreatic lesions have shown that activating *KRAS* mutations occur early during PanIN development, whereas alterations in tumor suppressor genes such as *CDKN2A/p16*, *TP53*, and *SMAD4/DPC4* are associated with progression toward high-grade PanIN and invasive PDAC [1,2]. Recent lineage-tracing and inducible genetic studies further suggest that pancreatic acinar cells can serve as a cellular origin of PanIN-like lesions and PDAC through acinar-to-ductal metaplasia (ADM) [3,4]. Thus, ADM, PanIN, and invasive PDAC are commonly evaluated as a pathological continuum of acinar-cell-associated pancreatic neoplastic progression.

Genetically engineered mouse models based on pancreas-specific activation of endogenous oncogenic *Kras* have provided a powerful framework for modeling PDAC in vivo. Among the most widely used models are those combining Cre-loxP-mediated activation of *Kras*^LSL-G12D^ with mutant or deleted *Trp53*, which generate invasive and metastatic pancreatic cancer that recapitulates key pathological features of human PDAC [5]. However, in many conventional *Kras*/*Trp53*-driven models, recombination is driven by developmental pancreatic promoters such as *Pdx1*-Cre or *Ptf1a*-Cre. Because these promoters are active in pancreatic progenitor domains during embryogenesis, oncogenic mutations are introduced broadly into pancreatic epithelial lineages rather than selectively into adult acinar cells [3,5]. This represents an important limitation for modeling sporadic human PDAC, which is thought to arise mainly from somatic mutations acquired in adult tissues. Therefore, models that permit temporally controlled oncogenic activation in the adult pancreas, particularly within the acinar lineage, are valuable for studying adult-onset pancreatic tumorigenesis.

Inflammatory injury is an important modifier in pancreatic cancer development. Clinically, chronic pancreatitis is associated with an increased risk of PDAC, and experimental studies have shown that pancreatic inflammation strongly promotes *Kras*-driven pancreatic neoplastic progression. Importantly, in adult-onset *Kras*-driven mouse models, progression to advanced PanIN and invasive PDAC is inefficient in the absence of experimentally induced pancreatitis, whereas pancreatitis strongly promotes neoplastic progression [6]. These findings support the concept that pancreatitis-induced inflammation provides a permissive environment for adult-onset *Kras*-driven pancreatic tumorigenesis. These effects have been observed after both acute and chronic pancreatitis, and mechanistic studies have implicated suppression of oncogene-induced senescence, increased *Kras* and effector expression, and STAT5-dependent acinar plasticity [7,8,9,10].

Cerulein-induced pancreatitis is widely used as an inflammatory stimulus in genetically engineered PDAC models. However, cerulein protocols generally require repeated injections, including regimens involving multiple intraperitoneal injections per day over consecutive days, such as seven hourly injections per day for two days, which is technically laborious and imposes procedural burden on both investigators and animals [6,11]. Therefore, a simpler and less labor-intensive pancreatitis-inducing protocol would be useful for modeling inflammation-associated PDAC progression. Recent work has also used cerulein-induced injury to examine molecular regulators of ADM and PanIN progression in *Kras*-driven models [12].

L-Lysine, a basic amino acid, has been reported to induce pancreatic acinar injury when administered at high doses. Early studies in rats showed that intraperitoneal injection of excess L-Lysine caused pancreatic acinar cell necrosis, increased serum amylase and lipase levels, and early mitochondrial swelling [13]. Subsequent mechanistic work demonstrated that intraperitoneal L-Lysine administration induces acute necrotizing pancreatitis and that early mitochondrial injury precedes trypsinogen activation and NF-κB activation [14]. More recent experiments using isolated rodent pancreatic acinar cells further showed that high concentrations of L-Lysine directly induce mitochondrial swelling, loss of mitochondrial membrane potential, ATP depletion, and necrotic acinar cell death [15].

However, single-dose L-Lysine-induced pancreatitis has not been systematically characterized for use in adult-onset, lineage-traced Ptf1a^CreER^-based Kras/Trp53 mouse models. In this study, we first characterized the dose–response relationship and temporal course of acute pancreatitis induced by a single L-Lysine administration in mice. We then applied this approach to an inducible adult-onset, lineage-traced *Ptf1a*^CreER^; *Kras*^LSL-G12D^; *Trp53*^LSL-R172H^; Rosa26-RFP model and documented the subsequent development of ADM, PanIN, invasive PDAC, and metastatic disease. This study establishes a streamlined, single-dose L-Lysine platform for inducing acute pancreatitis and tracking subsequent neoplastic progression in adult-onset Kras/Trp53-driven mice.

## 2. Materials and Methods

### 2.1. Animals

All animal experiments were performed in accordance with the institutional guidelines for animal experimentation at Kyoto University and were approved by the Animal Research Committee of Kyoto University (approval no. 20-138 and 23-193). Mice were housed under specific pathogen-free conditions in a temperature- and humidity-controlled facility with a 12 h light/dark cycle and were given standard laboratory chow and water ad libitum.

To induce oncogenic mutations and lineage labeling in adult pancreatic cells, *Ptf1a*^CreER/+^ mice were crossed with *Kras*^LSL-G12D/+^ mice, *Trp53*^LSL-R172H/+^ mice, and *Rosa26*-RFP reporter mice to generate *Ptf1a*^CreER/+^; *Kras*^LSL-G12D/+^; *Trp53*^LSL-R172H/+^; *Rosa26*-RFP mice. The official strain names and MGI identifiers were as follows: *Ptf1a*^CreER/+^: *Ptf1a*^tm2(cre/ESR1)Cvw^, MGI:3771322; *Kras*^LSL-G12D/+^: *Kras*^tm4Tyj^, MGI:2429948; *Trp53*^LSL-R172H/+^: *Trp53*^tm2Tyj^, MGI:3039263; and Rosa26-RFP: Gt(ROSA)26Sor^tm1Hjf^, MGI:3696099. Both male and female mice were used. Littermates lacking Cre recombinase or mice receiving vehicle injection were used as controls where applicable.

For the initial L-Lys dose–response and time-course experiments, 6-week-old male C57BL/6JmsSlc mice were used. To evaluate the strain-dependent tolerability of L-Lys, additional dose–response experiments were performed using 6-week-old male C57BL/6NCrSlc and BALB/cCrSlc mice. C57BL/6JmsSlc, C57BL/6NCrSlc, and BALB/cCrSlc mice were obtained from Shimizu Laboratory Supplies (Kyoto, Japan). For pancreatic cancer progression experiments, mice were treated with tamoxifen (TAM) at 4–8 weeks of age, followed by L-lysine (L-Lys) administration at 2 weeks after TAM treatment. Mice were analyzed at the indicated time points, typically between 2 and 6 months after L-Lys administration.

Mouse genotyping was performed by PCR using genomic DNA extracted from ear punches. Primer sequences are listed in Supplementary Table S1. Genotyping PCR was performed under the following standard cycling conditions unless otherwise specified: initial denaturation at 98 °C for 5 s; 40 cycles of denaturation at 98 °C for 10 s, annealing at 60 °C for 5 s, and extension at 68 °C for 1 s; followed by a final extension at 68 °C for 5 s. For *Kras* genotyping, touchdown PCR was performed as follows: initial denaturation at 98 °C for 5 s; 10 touchdown cycles of denaturation at 98 °C for 10 s, annealing from 65 °C to 60 °C with a decrease of 0.5 °C per cycle for 5 s, and extension at 68 °C for 1 s; followed by 30 cycles of denaturation at 98 °C for 10 s, annealing at 60 °C for 5 s, and extension at 68 °C for 1 s; and a final extension at 68 °C for 5 s.

Mice were monitored at least 3 times per week after tamoxifen and L-Lys administration, and daily when they showed signs of illness or tumor progression. Humane endpoints included body weight loss exceeding 20%, severe lethargy, hunched posture, impaired mobility, severe abdominal distention, inability to access food or water, respiratory distress, or other signs of severe distress as determined by investigators or veterinary staff.

### 2.2. Experimental Design

The study consisted of two main experimental components. First, L-Lys-induced acute pancreatitis was characterized in mice by dose–response and time-course analyses. In the initial dose–response study, 6-week-old male C57BL/6JmsSlc mice received a single intraperitoneal injection of physiological saline or L-Lys at 1.0, 1.5, 2.0, 2.5, or 3.0 g/kg body weight and were analyzed 24 h after injection. Because 3.0 g/kg L-Lys caused death within 24 h in preliminary experiments, this dose was not used for subsequent experiments. For the time-course study, mice received 2.0 g/kg L-Lys and were analyzed at 0, 2, 6, 12, 18, 24, and 48 h after injection, unless otherwise specified.

To examine the strain-dependent tolerability of L-Lys, separate dose-titration experiments were performed in 6-week-old male C57BL/6NCrSlc and BALB/cCrSlc mice. Mice received a single intraperitoneal injection of physiological saline or L-Lys at 2.0, 2.5, or 3.0 g/kg body weight. The primary endpoint was survival at 24 h after injection.

Second, the single-dose L-Lysine protocol was applied to *Ptf1a^CreER/+^*; *Kras^LSL-G12D/+^*; *Trp53^LSL-R172H/+^*; *Rosa26-RFP* mice to characterize the subsequent pathological course in an adult-onset, lineage-traced pancreatic cancer model. TAM was administered at 4–8 weeks of age to induce Cre-mediated recombination, resulting in activation of oncogenic *Kras*^G12D^ and mutant *Trp53*^R172H^ and simultaneous RFP lineage labeling in *Ptf1a*-expressing pancreatic cells. Two weeks after TAM treatment, mice received a single intraperitoneal injection of L-Lys at 2.0 g/kg. Pancreatic tissues and distant organs were harvested at 3–6 months after L-Lys administration, or at other indicated time points.

No formal sample-size calculation was performed because this was an exploratory protocol-development study. All mice meeting the prespecified genotype and treatment criteria were included in the analysis unless they met exclusion criteria, including incorrect genotype, failed injection, severe unrelated illness, or inadequate tissue fixation. Histological quantification was performed by investigators without blinding to the experimental groups.

For the 3–5-month post-L-Lys cohort, seven mice met the genotype and treatment criteria. Six mice were evaluable for pancreatic tumor formation, defined as tumor status determined by detailed histological analysis or by unequivocal macroscopic pancreatic tumor formation upon abdominal inspection at tissue harvest. Detailed histological analysis was performed in representative mice harvested at 3, 4, and 5 months after L-Lys administration. In the remaining evaluable mice, detailed histological assessment was not available because the pancreatic tissues had been allocated to other experimental purposes. One additional 5-month mouse was excluded from the tumor-formation assessment because macroscopic tumor status could not be determined before the entire pancreas was allocated to a separate experiment.

### 2.3. Tamoxifen (TAM) Administration

TAM (Sigma-Aldrich, T5648; St. Louis, MO, USA) was dissolved in corn oil (Wako, 032-07016; Osaka, Japan) at a concentration of 20 mg/mL by incubation with intermittent vortexing and protection from light. The solution was prepared freshly or stored at −30 °C for up to 4 weeks [16].

To induce CreER-mediated recombination, TAM was administered intraperitoneally at 4 mg per mouse per injection. In the standard protocol, mice received TAM every 1–2 days for a total of 3–5 injections. Mice were monitored for body weight and general condition after TAM administration.

### 2.4. L-Lysine (L-Lys)-Induced Pancreatitis

L-lysine (Tokyo Chemical Industry; L0129; Tokyo, Japan) was dissolved in sterile physiological saline to prepare a stock solution at 200 mg/mL, and the solution was sterilized using a 0.22-μm filter. L-Lys solution was prepared freshly on the day of injection.

For induction of acute pancreatitis, mice received a single intraperitoneal injection of L-Lys at the indicated dose. The standard dose used for acute pancreatitis characterization and subsequent cancer-progression experiments was 2.0 g/kg body weight. For the strain-comparison dose-titration experiments, C57BL/6NCrSlc and BALB/cCrSlc mice received physiological saline or L-Lys at 2.0, 2.5, or 3.0 g/kg using the same preparation and administration procedures. Control mice received an equivalent volume of sterile physiological saline. Injection volume was adjusted according to body weight. Mice were not fasted before L-Lys administration. After injection, mice were returned to clean cages and monitored for activity, body weight, abdominal symptoms, and survival.

For acute pancreatitis characterization, blood and pancreatic tissue were collected at 0, 2, 6, 12, 18, 24, and 48 h after L-Lys injection.

### 2.5. Blood Collection and Measurement of Serum Amylase and Lipase

Blood samples were collected at the indicated time points by tail-tip cutting or cardiac puncture under deep anesthesia. Blood was collected into Mini collect II tubes (Greiner Bio-One, 450533; Kremsmünster, Austria), and centrifuged at 3000 rpm for 10 min at 4 °C. Serum was transferred to new tubes and analyzed immediately or stored at −80 °C until use.

Serum amylase and lipase levels were measured by Unitech Co., Ltd. (Chiba, Japan).

### 2.6. Tissue Harvesting

Mice were euthanized at the indicated time points under deep anesthesia followed by cervical dislocation, in accordance with institutional animal care guidelines. The pancreas was carefully dissected to preserve its anatomical structure and was inspected macroscopically for swelling, atrophy, fibrosis, tumor formation, or dissemination. Any visible metastatic or disseminated lesions were also collected when present.

For paraffin histology, tissues were fixed in 4% paraformaldehyde at 4 °C for 12–18 h, dehydrated through graded ethanol, cleared in HistoClear (National-Diagnostics, HS200; Atlanta, GA, USA), and embedded in paraffin. Paraffin sections were cut at 4 μm thickness [16].

For mitochondrial analysis by immunofluorescence, pancreatic tissues were fixed promptly after harvesting to minimize postmortem artifacts.

### 2.7. Histological Assessment of Acute Pancreatitis

H&E-stained pancreatic sections were evaluated using a semiquantitative histological scoring system modified from previously published histological assessment methods [17,18,19]. Interstitial edema, inflammatory cell infiltration, and acinar cell necrosis were each graded on a scale from 0 to 3.

Edema was scored as follows: 0, absent; 1, focal widening of the interlobular spaces; 2, diffuse widening of the interlobular spaces; and 3, marked edema with disruption and separation of the acini. Inflammatory cell infiltration was scored as follows: 0, absent; 1, focal interstitial or periductal infiltration; 2, parenchymal infiltration involving <50% of the lobules; and 3, parenchymal infiltration involving ≥50% of the lobules. Acinar cell necrosis was scored as follows: 0, absent; 1, minimal focal necrosis involving <5% of the pancreatic parenchyma; 2, focal necrosis involving 5–10% of the pancreatic parenchyma; and 3, diffuse or extensive necrosis involving >10% of the pancreatic parenchyma.

For each mouse, ten systematically selected, non-overlapping fields at ×200 magnification were evaluated. The sampled fields were distributed across the pancreatic section to include both peripheral and central regions. Field-level scores were averaged separately for edema, inflammatory cell infiltration, and acinar cell necrosis to generate one mouse-level value for each parameter.

### 2.8. Transmission Electron Microscopy

As previously described, ultrastructural analysis of pancreatic tissues was performed using transmission electron microscopy (TEM) [20]. Briefly, mice were perfused with phosphate-buffered saline containing 4% paraformaldehyde and 2% glutaraldehyde. Pancreatic tissues were cut into approximately 1-mm^3^ pieces, immersed in the same fixative overnight, and post-fixed with osmium tetroxide. The specimens were subsequently dehydrated, embedded in resin, sectioned into ultrathin sections, and stained with uranyl acetate and lead citrate. The sections were examined using a transmission electron microscope (H-7650; Hitachi, Tokyo, Japan) at the Division of Electron Microscopic Study, Kyoto University. Pancreatic tissue specimens from a previously reported experimental cohort were used for this analysis [20]. The electron micrographs presented here were not included in the previous publication.

### 2.9. Regional Quantification of TOM20 Fluorescence Intensity

For regional quantification of TOM20 fluorescence, fixed-size regions of interest (ROIs; 100 × 100 μm) were placed within peripheral and central acinar regions of pancreatic sections. Peripheral ROIs were positioned within 150 μm of the pancreatic outer edge and entirely within the pancreatic parenchyma, whereas central ROIs were positioned at least 300 μm from the nearest outer edge. The outermost 20 µm of pancreatic tissue was excluded to minimize tissue-edge artifacts. Pancreatic ducts, blood vessels, islets, large stromal areas, tissue artifacts, and extrapancreatic regions were excluded. Mean TOM20 fluorescence intensity within each ROI was measured using the histogram function of the BZ-H4M Measurement Application (BZ-X Analyzer; Keyence, Osaka, Japan) and corrected by subtracting the mean background fluorescence measured in a tissue-free area. Multiple ROIs were averaged to generate one peripheral and one central value for each mouse.

### 2.10. Assessment of RFP Lineage-Labeling Efficiency by FACS

To evaluate the efficiency of tamoxifen-induced lineage labeling in adult pancreatic acinar cells, adult *Ptf1a*^CreER/+^; Rosa26-RFP mice were administered tamoxifen intraperitoneally at 4 mg per mouse per injection for a total of five injections. Three weeks after tamoxifen administration, pancreata were harvested and dissociated into single-cell suspensionsusing the Tumor Dissociation Protocol for Single Cell RNA Sequencing (Demonstrated Protocol CG000147, Rev. B; 10× Genomics, Pleasanton, CA, USA). Dissociated cells were stained with APC-conjugated anti-mouse CD326/EpCAM antibody (BioLegend, 118213; San Diego, CA, USA) and DAPI. Fluorescence-activated cell sorting was performed using a BD Symphony S6 cell sorter (BD Biosciences, San Jose, CA, USA). Debris was first excluded by FSC/SSC gating, and viable cells were selected as DAPI-negative cells. EpCAM-positive epithelial cells were then gated, and the proportion of RFP-positive cells within the live EpCAM-positive fraction was analyzed. Using this strategy, 96.8% of live EpCAM-positive pancreatic epithelial cells were RFP-positive, indicating highly efficient tamoxifen-induced lineage labeling in the adult *Ptf1a*-lineage pancreatic exocrine compartment.

### 2.11. Histology and Alcian Blue Staining

Paraffin sections were deparaffinized in HistoClear, rehydrated through graded ethanol, and stained with hematoxylin and eosin (H&E) according to standard protocols. H&E-stained sections were used to evaluate acute pancreatitis, acinar cell injury, acinar cell necrosis, inflammatory cell infiltration, acinar-to-ductal metaplasia (ADM), pancreatic intraepithelial neoplasia (PanIN), invasive pancreatic ductal adenocarcinoma (PDAC), and distant metastasis.

For Alcian Blue staining, sections were deparaffinized, rehydrated, immersed in 3% acetic acid, and stained with Alcian Blue solution, pH 2.5 (Muto Pure Chemicals, 4085-1; Tokyo, Japan), for 30 min. Sections were counterstained with Contrast Red Solution (KPL, 71-00-05, Gaithersburg, MD, USA), dehydrated, cleared, and mounted. Alcian Blue-positive mucinous lesions were used to identify ADM/PanIN-like epithelial lesions.

Histopathological evaluation was performed based on established criteria for murine pancreatic lesions. ADM was defined as duct-like epithelial structures arising within or replacing acinar lobules. PanIN lesions were defined as mucin-producing ductal epithelial lesions with variable degrees of epithelial atypia and papillary or flat architecture. PDAC was defined as invasive glandular carcinoma with stromal desmoplasia and/or invasion into adjacent pancreatic parenchyma or extrapancreatic tissues. When appropriate, PanIN lesions were classified as low-grade or high-grade according to epithelial atypia, nuclear stratification, papillary architecture, and loss of polarity.

### 2.12. Immunohistochemistry and Immunofluorescence

Immunohistochemistry and immunofluorescence were performed on paraffin sections. For paraffin sections, slides were deparaffinized, rehydrated, and subjected to antigen retrieval in 10 mM citrate buffer, pH 6.0, or Tris-EDTA buffer, pH 9.0, using a pressure cooker. Endogenous peroxidase activity was quenched with REAL Peroxidase-Blocking Solution (DAKO, S2023, Glostrup, Denmark) for 10 min when horseradish peroxidase-based detection was used. Sections were blocked with animal-free blocking solution (Protein Block: DAKO, X0909, Glostrup, Denmark) for 30 min at room temperature.

Primary antibodies were diluted in Immuno Shot blocking solution (Cosmo Bio, IS-P-500; Tokyo, Japan) and incubated at 4 °C overnight. After washing with PBS containing 0.05% Tween-20, sections were incubated with appropriate secondary antibodies for 1 h at room temperature. For immunohistochemistry, signals were detected using ImmPRESS HRP polymer reagents (Vector Laboratories, Newark, CA, USA) and 3,3′-diaminobenzidine (DAB; Vector Laboratories, 94010; Newark, CA, USA), followed by counterstaining with hematoxylin. For immunofluorescence, Alexa Fluor- or Cy3-conjugated secondary antibodies were used, and nuclei were counterstained with DAPI. Sections were mounted using Vectashield antifade mounting media (Vector Laboratories, H-1200; Newark, CA, USA).

The primary antibodies used in this study are listed in Supplementary Table S2.

The secondary antibodies used for immunofluorescence included donkey anti-rabbit Alexa Fluor 488 (Invitrogen, A21206; Waltham, MA, USA), donkey anti-rat Alexa Fluor 488 (Invitrogen, A21208; Waltham, MA, USA), donkey anti-guinea pig FITC (Millipore, AP193F; Burlington, MA, USA), donkey anti-rabbit Cy3 (Chemicon, AP182C; Tokyo, Japan), donkey anti-mouse Cy3 (Millipore, AP192C; Burlington, MA, USA), donkey anti-rat Cy3 (Millipore, AP189C; Burlington, MA, USA), donkey anti-guinea pig Cy3 (Millipore, AP193C; Burlington, MA, USA), and donkey anti-mouse Alexa Fluor 647 (Millipore, AP192SA6; Burlington, MA, USA), typically at 1:500 dilution. For HRP-based detection, ImmPRESS HRP horse anti-rabbit IgG (Vector Laboratories, MP-7401; Newark, CA, USA), horse anti-goat IgG (Vector Laboratories, MP-7405; Newark, CA, USA), or horse anti-mouse IgG (Vector Laboratories, MP-7422; Newark, CA, USA) was used according to the manufacturer’s instructions.

### 2.13. Microscopy and Image Acquisition

Bright-field images were acquired using a BZ-X800 fluorescence microscope (Keyence, Osaka, Japan). Fluorescence images were acquired using a BZ-X800 microscope or LSM900 confocal microscopes (Carl Zeiss, Jena, Germany). Exposure time, laser power, detector gain, and other acquisition parameters were kept constant within each staining experiment when quantitative comparison was performed.

Low-magnification whole-section images were used to assess lesion distribution and tumor burden. High-magnification images were used to evaluate cellular localization of RFP, CK19, E-cadherin, p44/42 MAPK, TOM20, and other markers.

### 2.14. Statistical Analysis

Statistical analyses were performed using GraphPad Prism version 11.0.2 (GraphPad Software, Boston, MA, USA). Data are presented as mean ± SEM unless otherwise indicated. For comparisons among multiple groups, one-way ANOVA followed by Holm– Šídák’s multiple-comparison test was used. Paired comparisons of peripheral and central TOM20 fluorescence intensity within the same mouse were performed using a two-tailed paired Student’s *t*-test. A *p* value < 0.05 was considered statistically significant. The number of biological replicates is indicated in each figure legend. Representative images were selected from at least three biologically independent samples unless otherwise stated.

### 2.15. Key Reagents and Materials

See Supplementary Table S3.

## 3. Results

### 3.1. Single-Dose L-Lysine Induces Acute Pancreatitis with Mitochondrial Injury

**Figure 1:**
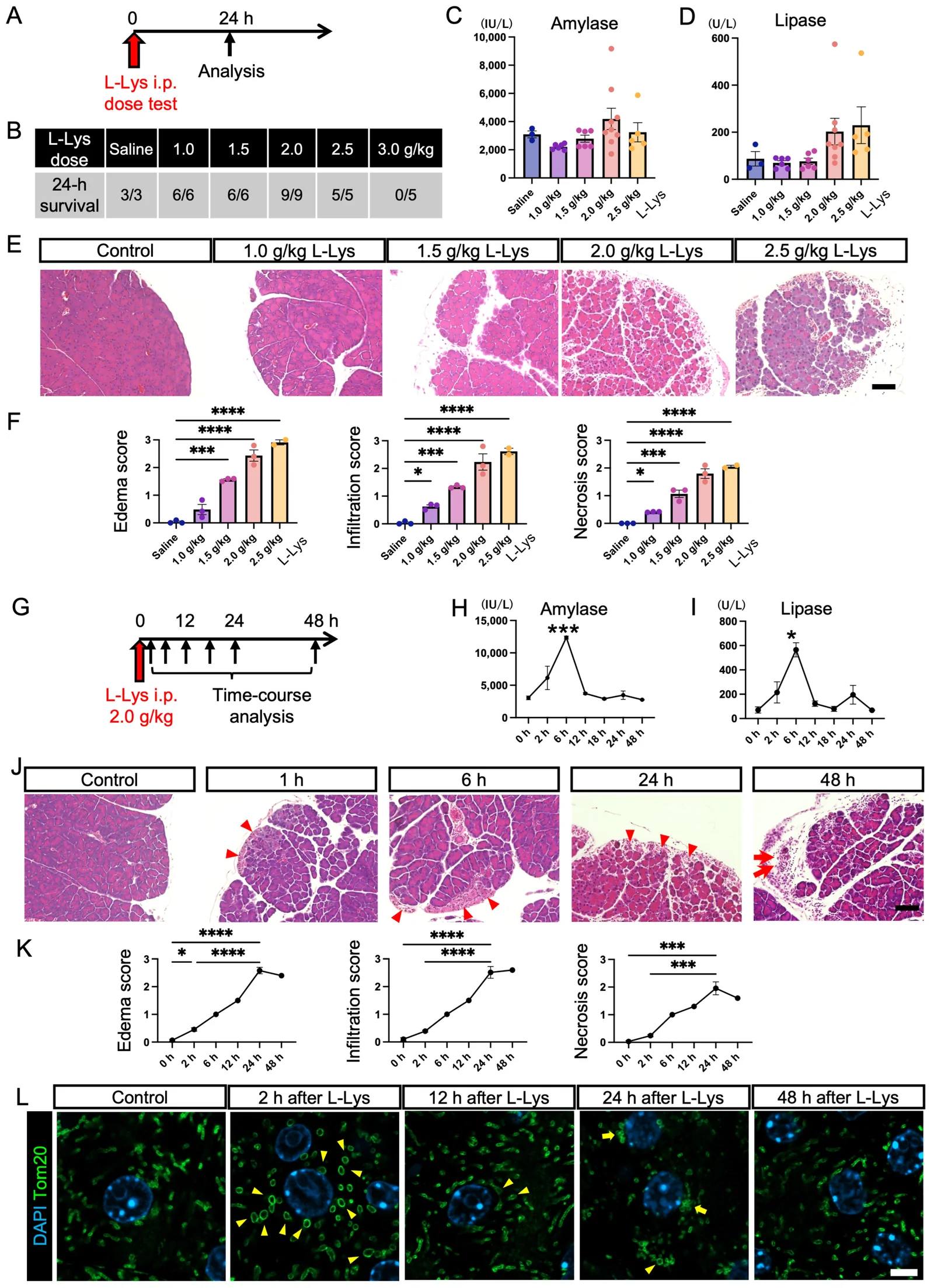
Single-dose L-Lysine induces acute pancreatitis with mitochondrial injury. (**A**) Experimental scheme for the L-Lysine dose–response study. Mice received a single intraperitoneal injection of physiological saline or L-Lysine at the indicated dose and were analyzed 24 h after injection. (**B**) Twenty-four-hour survival after L-Lysine administration. All mice treated with 1.0–2.5 g/kg L-Lysine survived for 24 h, whereas all mice treated with 3.0 g/kg L-Lysine died within 24 h. (**C**,**D**) Serum amylase (**C**) and lipase (**D**) levels 24 h after physiological saline or L-Lysine administration. Data are presented as mean ± SEM. *n* = 3–9 mice per group, as indicated. (**E**) Representative H&E-stained pancreatic sections 24 h after physiological saline or L-Lysine administration. L-Lysine induced acinar cell injury, with more evident histological damage at 2.0–2.5 g/kg. (**F**) Dose–response histological scores for edema, inflammatory cell infiltration, and acinar cell necrosis. All three parameters increased with the L-Lysine dose. * *p* < 0.05, *** *p* < 0.001, and **** *p* < 0.0001 vs. saline; ordinary one-way ANOVA with Holm-Šídák’s multiple comparisons test. *n* = 3 for the saline, 1.0–2.0 g/kg groups and *n* = 2 for the 2.5 g/kg group. (**G**) Experimental scheme for the time-course analysis after a single intraperitoneal injection of 2.0 g/kg L-Lysine. (**H**,**I**) Time-course changes in serum amylase (**H**) and lipase (**I**) levels after 2.0 g/kg L-Lysine administration. Serum amylase and lipase levels increased rapidly after L-Lysine administration and declined thereafter. Data are presented as mean ± SEM. *n* = 2–6 mice per group. * *p* < 0.05; *** *p* < 0.001 vs. 0 h. (**J**) Representative H&E-stained pancreatic sections at the indicated time points after 2.0 g/kg L-Lysine administration. Red arrowheads and arrows indicate acinar cell injury and acinar cell necrosis, respectively. (**K**) Time-course histological scores for edema, inflammatory cell infiltration, and acinar cell necrosis. Edema and inflammatory cell infiltration increased over time, whereas acinar cell necrosis was highest at 24 h. * *p* < 0.05, *** *p* < 0.001, **** *p* < 0.0001; ordinary one-way ANOVA with Holm-Šídák’s multiple comparisons test, *n* = 3 at 0, 2 and 24 h. The 6, 12 and 48 h groups were not included in the statistical analysis because only one mouse was available at each time point. (**L**) Immunofluorescence staining for TOM20 and DAPI showing mitochondrial alterations after L-Lysine administration. Enlarged and rounded TOM20-positive mitochondrial profiles were prominent at 2 h and were occasionally observed at 12 and 24 h after L-Lysine administration (arrowheads). At 24 h, TOM20-positive mitochondria exhibited focal accumulation adjacent to the nuclei and an altered intracellular distribution (arrows). These abnormalities were followed by partial recovery at 48 h. Control mice received physiological saline and were analyzed 24 h after injection. Scale bars: 100 µm (**E**,**J**) and 5 µm (**L**).

We first examined whether a single intraperitoneal injection of L-Lysine could induce acute pancreatitis in mice and determined an appropriate dose for subsequent cancer-progression experiments. Mice received physiological saline or L-Lysine at 1.0, 1.5, 2.0, 2.5, or 3.0 g/kg body weight and were analyzed 24 h after injection (Figure 1A). All mice treated with 1.0–2.5 g/kg L-Lysine survived for 24 h, whereas all mice treated with 3.0 g/kg L-Lysine died within 24 h (Figure 1B). Serum amylase and lipase measurements supported pancreatic injury at higher non-lethal doses, although enzyme levels varied among individual mice (Figure 1C,D). Histological analysis revealed acinar cell injury after L-Lysine administration, with more evident pancreatic damage at 2.0–2.5 g/kg (Figure 1E). Semiquantitative histological scoring of interstitial edema, inflammatory cell infiltration, and acinar cell necrosis further showed dose-dependent increases in all three parameters, with pronounced pancreatic injury at L-Lysine doses of 2.0 and 2.5 g/kg (Figure 1F). Together with the absence of 24 h mortality at 2.0 g/kg, these findings supported the selection of 2.0 g/kg as the standard dose for the subsequent time-course and experiments using the Kras/Trp53-driven pancreatic cancer model. During post-injection monitoring, mice treated with 2.0 g/kg L-Lysine showed no apparent body weight loss, in addition to complete 24 h survival, consistent with previous reports in rats [13,14].

To assess the influence of genetic background on L-Lys tolerability, we performed additional 24 h dose-titration experiments using C57BL/6NCrSlc mice, representing a distinct C57BL/6 substrain commonly used in genetically engineered mouse models, and BALB/cCrSlc mice, representing a more genetically divergent inbred strain (Supplementary Figure S1). All C57BL/6NCrSlc and BALB/cCrSlc mice survived for 24 h after 2.0 g/kg L-Lys administration (4/4 per strain). Survival at 2.5 and 3.0 g/kg was 3/4 and 0/3 in C57BL/6NCrSlc mice and 4/4 and 2/4 in BALB/cCrSlc mice, respectively, indicating strain-dependent lethality at higher doses.

We next characterized the time course of pancreatitis after a single injection of 2.0 g/kg L-Lysine (Figure 1G). Serum amylase and lipase levels increased rapidly after L-Lysine administration, both peaking at 6 h after injection, with approximately 4-fold and 8-fold increases over baseline, respectively, before declining toward later time points (Figure 1H,I). Histological analysis showed early acinar cell injury, followed by focal acinar cell necrosis during the later phase of pancreatitis (Figure 1J).

Semiquantitative histological scoring showed that pancreatic morphological injury remained evident after serum amylase and lipase levels had begun to decline. Edema and inflammatory cell infiltration remained evident at later time points, whereas acinar cell necrosis was most prominent at 24 h and remained detectable at 48 h (Figure 1K). These findings indicate that the biochemical and histopathological manifestations of L-Lysine-induced pancreatitis exhibit distinct temporal profiles. Macroscopically, L-Lysine-treated pancreata showed edema-like changes compared with saline-treated controls, consistent with acute pancreatitis (Supplementary Figure S2).

Because L-Lysine-induced pancreatitis has been associated with early mitochondrial injury [13,14,15], we examined mitochondrial morphology by immunofluorescence staining for TOM20, a mitochondrial outer membrane marker. In saline-treated control pancreata, TOM20 staining showed fine punctate to tubular mitochondrial patterns in acinar cells. By 2 h after L-Lysine administration, TOM20-positive mitochondrial structures became enlarged and rounded, indicating early disruption of mitochondrial organization (Figure 1L). At later time points, TOM20 staining showed partial restoration of mitochondrial organization, consistent with the transient nature of L-Lysine-induced mitochondrial injury.

Transmission electron microscopy further confirmed mitochondrial ultrastructural abnormalities after L-Lysine administration (Supplementary Figure S3). Mitochondrial swelling, reduced electron density, and cristae disruption were observed at 24 h, followed by the appearance of mitophagosome-like structures containing degenerating mitochondrial material at 48 h.

To quantitatively assess the regional distribution of TOM20 alterations, we compared mean TOM20 fluorescence intensity between peripheral and central acinar regions. In saline-treated pancreata, TOM20 fluorescence intensity did not differ significantly between the two regions. At 2 h after L-Lysine administration, however, mean TOM20 fluorescence intensity was significantly higher in the peripheral region than in the central region (Supplementary Figure S4). These findings demonstrate that the early TOM20 alterations induced by L-Lysine are spatially heterogeneous and are particularly prominent in the peripheral pancreatic region. Collectively, these findings indicate that a single intraperitoneal injection of 2.0 g/kg L-Lysine induces sublethal acute pancreatitis accompanied by rapid, transient, and spatially heterogeneous mitochondrial alterations.

### 3.2. Efficient Induction of Oncogenic Kras/Trp53 Mutations and Lineage Labeling in Adult Ptf1a-Lineage Pancreatic Cells

**Figure 2:**
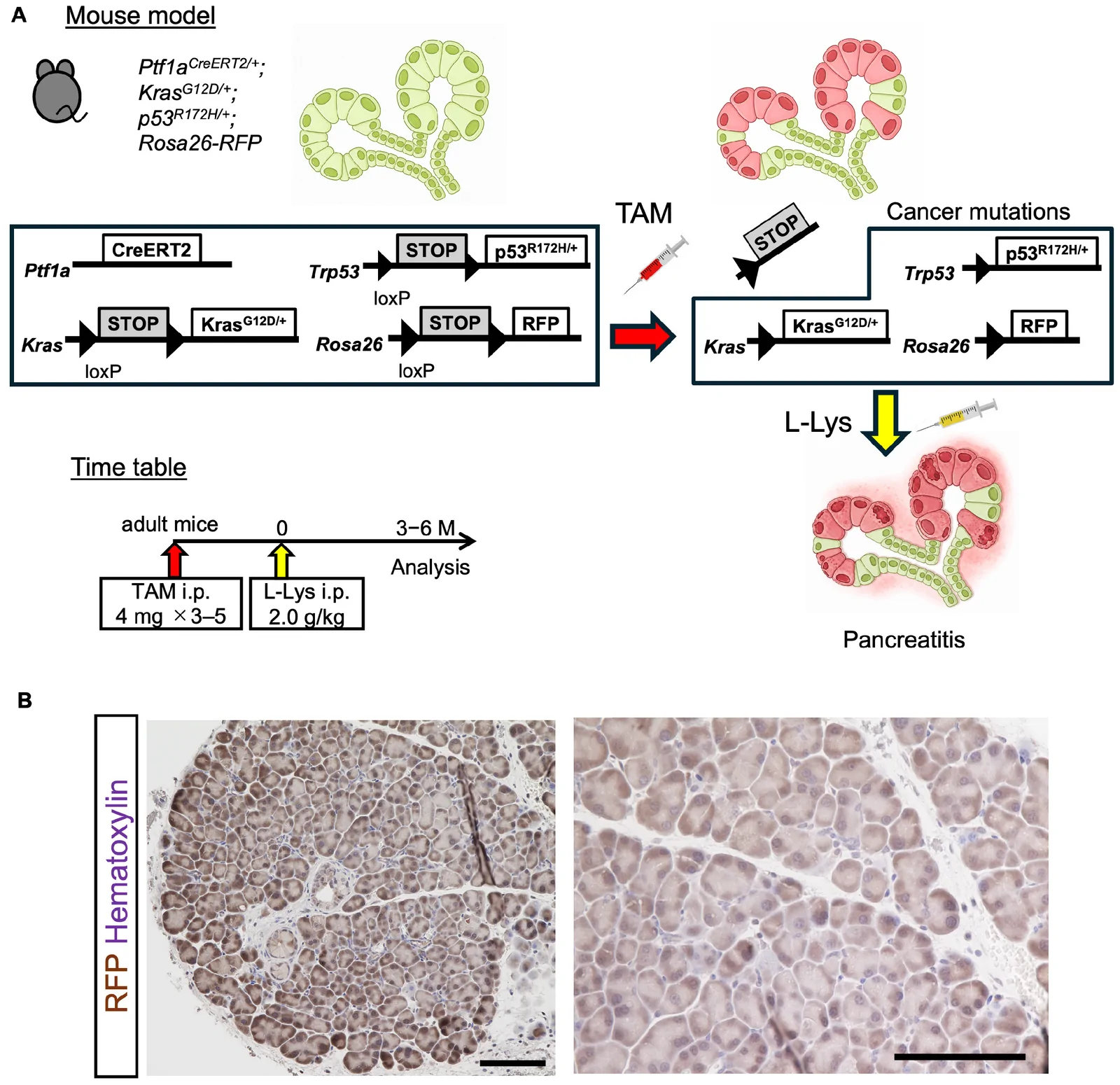
Experimental design of the L-Lysine-induced pancreatitis model in *Ptf1a*-CreER; *Kras*/*Trp53*/RFP mice. (**A**) Schematic representation of the inducible pancreatic cancer model. *Ptf1a*^CreER^; *Kras*^LSL-G12D^; *Trp53*^LSL-R172H^; Rosa26-RFP were used to induce oncogenic *Kras*^LSL-G12D^ and mutant *Trp53*^LSL-R172H^ expression, together with RFP lineage labeling, in *Ptf1a*-expressing pancreatic cells. Adult mice received intraperitoneal tamoxifen injections at 4 mg per injection for a total of 3–5 injections. Two weeks after tamoxifen administration, mice received a single intraperitoneal injection of L-Lysine at 2.0 g/kg and were analyzed 3–6 months after L-Lysine administration. Red acinar cells indicate TAM-recombined cells with induced Kras/Trp53 mutations, whereas green cells are unrecombined. (**B**) Representative RFP immunostaining with hematoxylin counterstaining after tamoxifen-induced recombination, showing efficient RFP lineage labeling in the adult pancreatic acinar compartment. Scale bars: 100 µm.

To apply the single-dose L-Lysine protocol in an adult-onset pancreatic cancer model, we used *Ptf1a*^CreER^; *Kras*^LSL-G12D^; *Trp53*^LSL-R172H^; Rosa26-RFP mice. In this model, tamoxifen administration induces Cre-loxP recombination in *Ptf1a*-expressing pancreatic acinar cells, resulting in activation of oncogenic *Kras*^G12D^ and mutant *Trp53*^R172H^ together with RFP lineage labeling (Figure 2A). Adult mice received intraperitoneal tamoxifen injections at 4 mg per injection for a total of 3–5 injections. Two weeks after tamoxifen treatment, mice received a single intraperitoneal injection of L-Lysine at 2.0 g/kg and were analyzed 3–6 months after L-Lysine administration (Figure 2A).

RFP immunostaining showed broad RFP labeling in the adult pancreatic acinar compartment, involving more than 90% of acinar cells by histological assessment (Figure 2B). In addition, FACS analysis of dissociated pancreatic epithelial cells from tamoxifen-treated *Ptf1a*^CreER^; Rosa26-RFP mice demonstrated that 96.8% of live EpCAM-positive pancreatic epithelial cells were RFP-positive (Supplementary Figure S5). Thus, FACS analysis confirmed efficient RFP labeling within the live EpCAM-positive pancreatic epithelial fraction, while histological analysis confirmed broad labeling of acinar cells. Together, these data support efficient tamoxifen-induced recombination in adult *Ptf1a*-lineage exocrine cells, supporting the use of this system for temporally controlled, adult-stage oncogenic Kras/Trp53 activation together with lineage tracing of pancreatic neoplastic lesions.

### 3.3. Multifocal Pancreatic Neoplastic Lesions Observed After Single-Dose L-Lysine-Induced Pancreatitis

**Figure 3:**
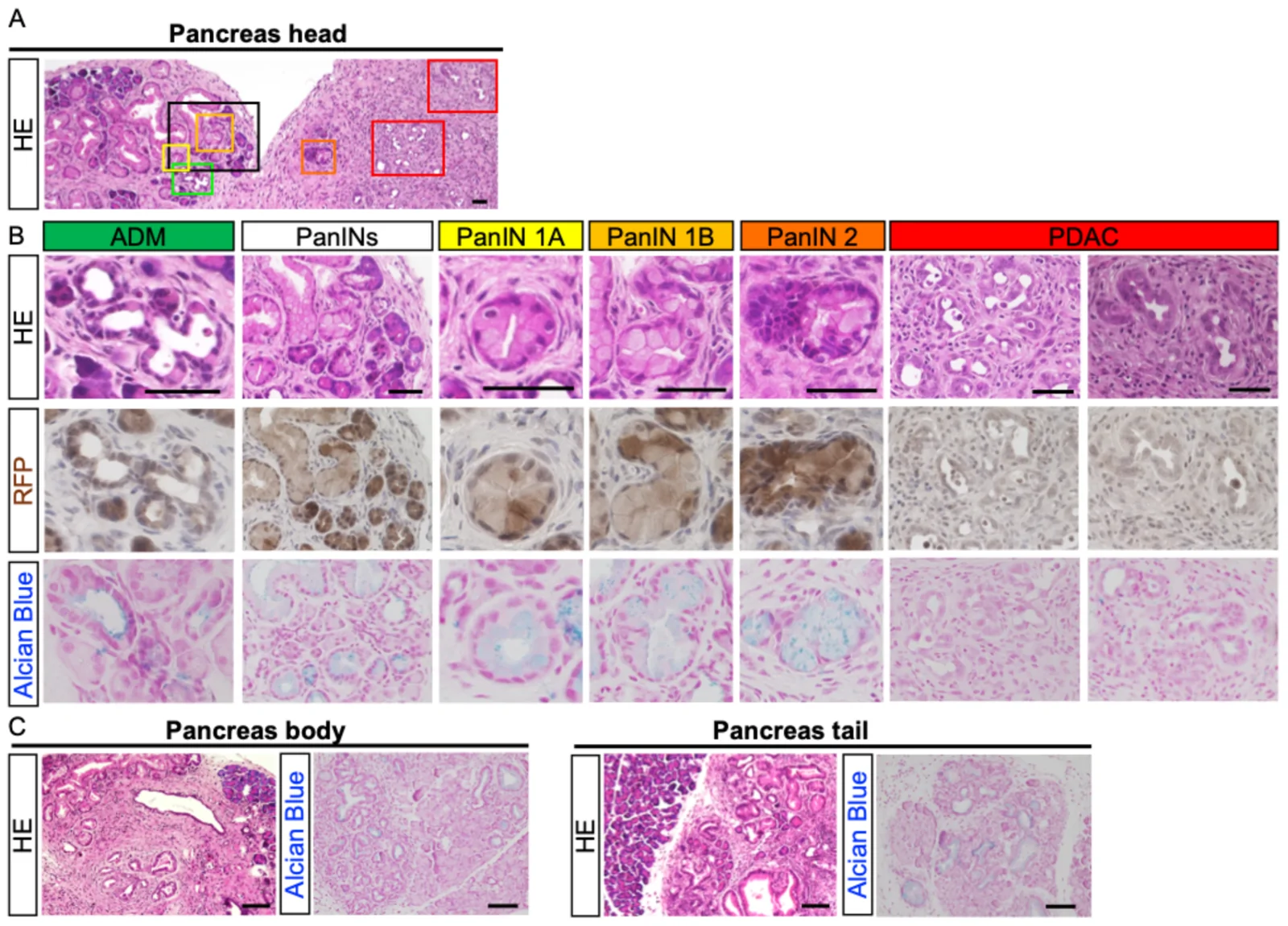
Multifocal pancreatic neoplastic lesions after the single-dose L-Lysine protocol in Kras/Trp53-driven mice. (**A**) Representative low-magnification H&E-stained section of the pancreatic head from an L-Lysine-treated *Ptf1a*^CreER^; *Kras*^LSL-G12D^; *Trp53*^LSL-R172H^; Rosa26-RFP mouse. Multiple pancreatic neoplastic lesions, including acinar-to-ductal metaplasia (ADM), pancreatic intraepithelial neoplasia (PanIN), and invasive pancreatic ductal adenocarcinoma (PDAC), coexisted within the same pancreas. (**B**) Representative high-magnification images of ADM, PanIN lesions, and PDAC. H&E staining shows the histological spectrum from ADM and low-grade PanIN to PDAC. RFP immunostaining confirms that these epithelial lesions arose from recombined *Ptf1a*-lineage cells. Alcian Blue staining highlights mucin-producing ADM/PanIN-like epithelial lesions. (**C**) Representative H&E and Alcian Blue staining of the pancreatic body and tail from the same mouse. Multifocal ADM/PanIN-like mucinous lesions were observed across the pancreatic head, body, and tail after a single episode of L-Lysine-induced pancreatitis. The colored boxes in panel A indicate the regions shown at higher magnification in panel B: green, ADM; black, PanINs; yellow, PanIN-1A; orange, PanIN-1B/PanIN-2; and red, PDAC. Scale bars: 50 µm (**A**,**B**) and 100 µm (**C**).

We then examined pancreatic tissues from L-Lysine-treated *Ptf1a*^CreER^; *Kras*^LSL-G12D^; *Trp53*^LSL-R172H^; Rosa26-RFP mice. At 3 months after L-Lysine administration, histological analysis of the pancreatic head revealed multiple neoplastic lesions within the same pancreas, including acinar-to-ductal metaplasia (ADM), pancreatic intraepithelial neoplasia (PanIN), and invasive pancreatic ductal adenocarcinoma (PDAC) (Figure 3A). These lesion categories were interpreted according to established PanIN/PDAC progression criteria and acinar-cell-associated ADM/PanIN frameworks [1,3,4]. Higher-magnification analysis demonstrated a continuous spectrum from ADM and low-grade PanIN lesions to more advanced PanIN lesions and PDAC (Figure 3B). RFP immunostaining confirmed that these epithelial lesions arose from recombined *Ptf1a*-lineage acinar cells, whereas Alcian Blue staining highlighted mucin-producing ADM/PanIN lesions, supporting the development of precancerous pancreatic epithelial lesions after L-Lysine-induced pancreatitis (Figure 3B).

The neoplastic lesions were not restricted to a single anatomical region. Histological analysis together with Alcian Blue staining showed multifocal ADM/PanIN-like mucinous lesions across the pancreatic head, body, and tail in the same mouse (Figure 3C). In the cohort analyzed between 3 and 5 months after L-Lysine administration, pancreatic tumor formation was evident in all evaluable mice (6/6). Detailed histological characterization of representative mice analyzed at 3, 4, and 5 months confirmed pancreatic neoplastic progression, including multifocal ADM, PanIN lesions, and PDAC (Figure 3 and Supplementary Figure S6). Gross examination of a representative 5-month mouse also revealed advanced pancreatic tumor formation (Supplementary Figure S7).

In representative lesions from the 4- and 5-month samples, RFP immunostaining confirmed the recombined *Ptf1a*-lineage origin of epithelial cells, CK19 and E-cadherin staining demonstrated ductal epithelial differentiation, and phospho-p44/42 MAPK staining indicated activation of the MAPK pathway downstream of oncogenic *Kras* [5] (Supplementary Figure S6). These findings document broad and multifocal pancreatic neoplastic pathology in the L-Lysine-treated cohort during 3–5 months of follow-up. Although this treated cohort was not designed for direct comparison with no-pancreatitis controls, tumor formation was evident in all six evaluable mice.

### 3.4. Invasive PDAC with Peritoneal Metastasis After L-Lysine Administration

**Figure 4:**
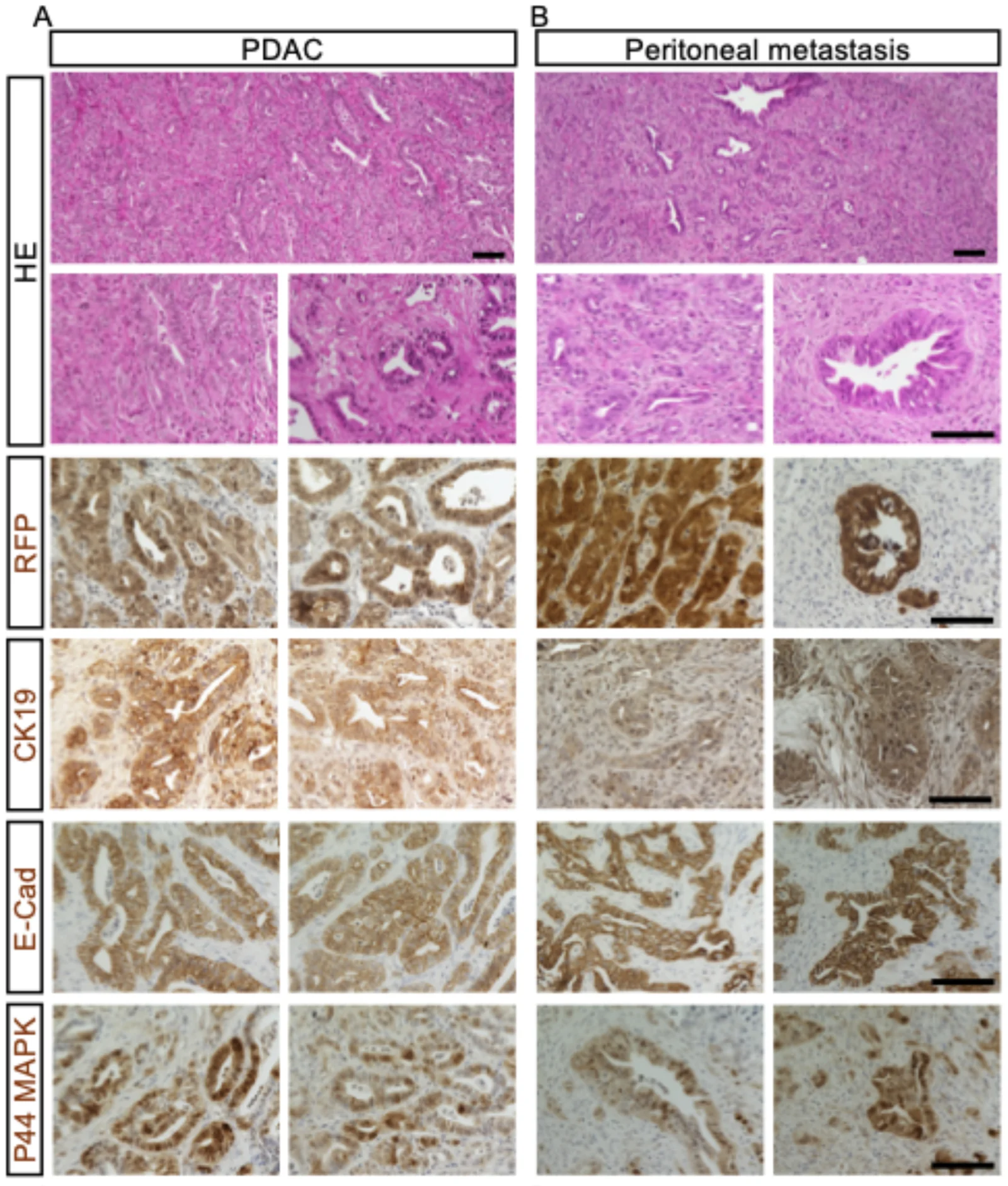
Invasive PDAC and peritoneal metastasis at 6 months after L-Lysine administration. (**A**) Representative images of the primary pancreatic tumor from an L-Lysine-treated *Ptf1a*^CreER^; *Kras*^LSL-G12D^; *Trp53*^LSL-R172H^; Rosa26-RFP mouse at 6 months after L-Lysine administration. H&E staining shows invasive pancreatic ductal adenocarcinoma with stromal desmoplasia. RFP immunostaining confirms the recombined *Ptf1a*-lineage origin of the tumor epithelial cells. CK19 and E-cadherin staining demonstrate ductal epithelial differentiation, and phospho-p44/42 MAPK staining indicates activation of MAPK signaling. (**B**) Representative images of a peritoneal metastatic lesion obtained from the same mouse shown in (**A**). The metastatic lesion showed histological and immunohistochemical features similar to the primary PDAC, including RFP lineage labeling, CK19 expression, E-cadherin expression, and phospho-p44/42 MAPK activation. Scale bars: 100 µm.

We next evaluated advanced lesions at later time points after L-Lysine-induced pancreatitis. Histological examination at 6 months after L-Lysine administration revealed invasive PDAC with stromal desmoplasia (Figure 4A). RFP immunostaining confirmed the recombined *Ptf1a*-lineage origin of the tumor epithelial cells. The primary tumor expressed CK19 and E-cadherin, consistent with ductal epithelial differentiation, and showed phospho-p44/42 MAPK staining, indicating activation of MAPK signaling downstream of oncogenic Kras [5] (Figure 4A).

Importantly, advanced intraperitoneal disseminated disease was observed in two mice at 6 months after L-Lysine administration. In the representative mouse shown in Figure 4, a peritoneal metastatic lesion obtained from the same animal as the primary pancreatic tumor showed histological and immunohistochemical features similar to those of the primary PDAC, including RFP lineage labeling, CK19 expression, E-cadherin expression, and phospho-p44/42 MAPK activation (Figure 4B). In another mouse that died around the 6-month endpoint, necropsy revealed conglomeration of intra-abdominal organs into a tumor-associated mass occupying much of the abdominal cavity, a finding consistent with extensive intraperitoneal tumor dissemination, although histological analysis was not available. Given the advanced disseminated disease and rapid clinical deterioration observed at this stage, analyses beyond 6 months were not pursued for animal welfare reasons.

Together, these findings establish the technical feasibility of combining a single-dose L-Lysine pancreatitis protocol with adult-stage *Ptf1a*-lineage *Kras/Trp53* activation and define the pathological outcomes observed in the treated cohort.

## 4. Discussion

In this study, we characterized a dose-optimized, single-dose L-Lysine protocol for inducing synchronized acute pancreatitis and applied it to an adult-onset, lineage-traced *Ptf1a*-CreER; *Kras/Trp53* pancreatic cancer model. A single intraperitoneal injection of 2.0 g/kg L-Lysine induced sublethal acute pancreatitis characterized by increased serum pancreatic enzymes, quantitatively assessed edema, inflammatory cell infiltration, acinar cell necrosis, and early mitochondrial alterations. Following application of this protocol to tamoxifen-treated *Ptf1a*-lineage *Kras/Trp53* mice, the treated animals exhibited multifocal ADM, PanIN lesions, invasive PDAC, and peritoneal dissemination during several months of follow-up.

An important contribution of the present study is the integrated characterization of a single-dose L-Lysine pancreatitis protocol in an adult-onset, lineage-traced pancreatic cancer model. The study defines the effective dose, temporal injury profile, quantitative histological severity, strain-dependent tolerability, and early mitochondrial alterations, while also documenting the subsequent occurrence of multifocal ADM and PanIN lesions, invasive PDAC, and peritoneal metastasis. This integrated framework provides practical information for the reproducible application of L-Lysine-induced pancreatic injury to genetically engineered mouse models and for the longitudinal assessment of injury-associated neoplastic progression.

Genetically engineered mouse models have been indispensable for studying PDAC initiation and progression. Conventional models based on pancreas-specific activation of oncogenic *Kras*, particularly in combination with mutant or deleted *Trp53*, reproduce key pathological features of human PDAC, including PanIN formation, invasive carcinoma, and metastatic progression [5]. However, many widely used models rely on developmental Cre drivers such as *Pdx1*-Cre or *Ptf1a*-Cre, in which oncogenic mutations are induced broadly in pancreatic epithelial progenitor domains during embryogenesis. Although these models are powerful and highly informative, this developmental timing differs from sporadic human PDAC, which is generally thought to arise from somatic mutations acquired in adult tissues. In this regard, inducible adult-stage models are valuable because they permit temporal control of oncogene activation and allow pancreatic injury to be introduced after genetic recombination.

Previous studies have shown that mature acinar cells remain susceptible to oncogenic *Kras*-driven transformation and can give rise to PanIN lesions through acinar-to-ductal metaplasia, although oncogenic *Kras* activation alone in adult acinar cells was insufficient to generate invasive carcinoma in that setting [3,21]. This distinction is important because it suggests that adult acinar cells can initiate precursor lesions, but that additional tumor-suppressor alterations and/or injury-associated microenvironmental cues are required for efficient progression to invasive PDAC. In adult *Kras*-driven models, pancreatic inflammation has also been shown to provide a critical permissive environment for progression toward PDAC [6]. Our model builds on these concepts by combining adult-stage *Ptf1a*-lineage oncogenic activation, mutant *Trp53*, and a defined, single-dose pancreatic injury. Recent inducible models further demonstrate that the pre-existing acinar-cell transcriptional state, including loss of *Rbpj*, can substantially influence sensitivity to oncogenic *Kras* and PanIN initiation [22].

A major practical advantage of the present protocol is its simplicity. Cerulein-induced pancreatitis is one of the most commonly used approaches to induce experimental pancreatic injury, but it typically requires repeated injections, including multiple hourly intraperitoneal injections for acute pancreatitis and repeated sessions over weeks for recurrent or chronic pancreatitis models [11]. Such repeated procedures are labor-intensive and introduce cumulative variability related to handling, injection timing, injection site, and technical execution. In contrast, L-Lysine-induced pancreatitis can be initiated by a single intraperitoneal injection, thereby providing a clearly defined starting point for subsequent time-course analysis. This feature may be particularly useful for large experimental cohorts, genetically complex mouse models, intervention studies, and omics-based analyses that require synchronized tissue collection.

The single-dose nature of the L-Lysine protocol is also relevant from the perspective of animal welfare. Repeated injection-based pancreatitis protocols impose procedural burden on animals through repeated restraint, handling, and needle puncture. Reducing the number of interventions is consistent with the principle of refinement within the 3Rs framework and with current expectations for reporting and improving animal experiments [23]. In the present study, 2.0 g/kg L-Lysine induced pancreatic injury without apparent short-term mortality or body weight loss, whereas 3.0 g/kg caused death within 24 h, indicating the importance of careful dose optimization. In addition, advanced disseminated disease and rapid clinical deterioration were observed at approximately 6 months after L-Lysine administration; therefore, analyses beyond this stage were not pursued for animal welfare reasons. Thus, this model may reduce procedural burden during pancreatitis induction while still producing advanced pathological endpoints within a relatively short observation period for an adult-onset pancreatic cancer model.

Beyond procedural convenience, L-Lysine-induced pancreatitis may also provide a biologically distinct injury context. Unlike cerulein-induced pancreatitis, which represents a secretagogue hyperstimulation model, L-Lysine-induced pancreatitis is characterized by early mitochondrial acinar injury [13,14,15], consistent with the TOM20 and ultrastructural abnormalities observed in this study. Recent studies indicate that mitochondrial and redox stress can influence pancreatic epithelial plasticity: mitochondrial ROS and loss of NADPH-producing enzymes have been linked to ADM/PanIN formation and tumor progression [24,25,26]. However, these effects may be stage dependent [27], and whether transient L-Lysine-induced mitochondrial injury is causally required for subsequent inflammatory and neoplastic responses remains unresolved.

Separately, supportive evidence for an injury-associated tumor-promoting effect comes from our recent study using an independent, exocrine-specific *Elastase-CreER*-based *Kras/Trp53* model [20]. In that study, mice without L-Lysine showed essentially no detectable Alcian Blue-positive neoplastic area at 3 months (*n* = 6 in total), whereas L-Lysine-treated mice developed PanIN lesions by 3 weeks and large fibrotic pancreatic tumors by 2 months. These findings support the biological plausibility of an injury-associated effect in that model but cannot substitute for a matched control in the present *Ptf1a-CreER* cohort, because the Cre drivers and resulting tumor kinetics may differ and the published comparison was not a contemporaneous control for the current study. Recent comparative analyses have also shown that the choice of inducible acinar-cell Cre driver, including *Ptf1a*CreERT-, *Mist1*CreERT-, and Elastase-CreERT-based systems, can alter the molecular and histological response to oncogenic *Kras* and cerulein-induced injury [28].

Despite its procedural simplicity and distinct mitochondrial injury profile, L-Lysine-induced pancreatitis should be regarded as complementary to, rather than universally superior to, cerulein-induced pancreatitis. The two models initiate pancreatic injury through different mechanisms and may therefore be suited to different experimental questions. Future head-to-head comparisons between L-Lysine and cerulein protocols in the same genetic background will be required to determine whether these injury modalities differ in the kinetics, penetrance, immune microenvironment, stromal response, and metastatic behavior of PDAC. A recent cerulein-associated *Kras*-driven GEMM study further demonstrated that lesion progression can be accompanied by marked immunostromal remodeling, including myofibroblast activation, IL-6–STAT3 signaling, and neutrophil infiltration [29].

The coexistence of ADM, PanIN lesions, and invasive PDAC within the same pancreas indicates that the pathological process observed in the treated cohort was spatially heterogeneous rather than a uniform linear sequence in which each lesion stage appeared at a completely distinct time point. Multifocal ADM/PanIN-like lesions were distributed across the pancreatic head, body, and tail. The RFP-positive epithelial origin, ductal marker expression, and MAPK activation further support the derivation of these lesions from recombined Ptf1a-lineage cells undergoing oncogenic Kras-associated ductal reprogramming.

The development of peritoneal dissemination by 6 months after L-Lysine administration further indicates that this protocol may be useful not only for studying ADM/PanIN formation and local invasion but also for investigating advanced tumor progression and intraperitoneal dissemination. Given that metastatic and disseminated disease is a major cause of mortality in PDAC, a model that progresses from adult-onset oncogenic activation to invasive and disseminated cancer within several months may provide a useful platform for therapeutic and mechanistic studies.

This study has several limitations: First, the present study was primarily designed to characterize the single-dose L-Lysine protocol and the pathological outcomes observed after its application to adult Ptf1a-lineage Kras/Trp53-driven mice. A contemporaneous genotype-matched no-pancreatitis cohort was not included; therefore, the extent to which L-Lysine-induced pancreatic injury altered tumor incidence, latency, or metastatic progression relative to the untreated course of this model remains to be determined.

Second, the number of mice analyzed for long-term tumor outcomes was limited. Although pancreatic tumor formation was evident in all evaluable mice analyzed between 3 and 5 months after L-Lysine administration, detailed histological assessment was performed in representative animals, and the study was not designed as a formal penetrance or survival analysis.

Third, the present study did not include a direct head-to-head comparison with cerulein-induced pancreatitis. Therefore, differences between L-Lysine and cerulein in tumor kinetics, inflammatory responses, stromal remodeling, immune-cell composition, and metastatic behavior remain unknown.

Fourth, L-Lysine tolerability varied according to genetic background at higher doses. Although 2.0 g/kg was tolerated during the 24 h observation period in the three strains examined, equivalent survival does not demonstrate equivalent pancreatic injury. Preliminary dose titration together with biochemical and histological validation is therefore recommended for genetically divergent or mixed-background mice.

Fifth, TOM20 staining and ultrastructural analyses documented early mitochondrial alteration after L-Lysine administration, but the present study did not directly test whether these alterations are causally required for subsequent inflammatory or neoplastic responses. Appropriately timed pharmacological and genetic perturbations of mitochondrial function and redox homeostasis will be required to establish causality.

## 5. Conclusions

We describe a dose-optimized, single-dose L-Lysine protocol that induces synchronized acute pancreatitis with early mitochondrial alterations and can be combined with adult *Ptf1a*-lineage, *Kras*/*Trp53*-driven mice. This model combines practical simplicity, reduced procedural burden, adult-stage oncogenic activation, lineage tracing, and occurrence of multifocal precursor lesions, invasive PDAC, and peritoneal dissemination. In addition to serving as a practical pancreatitis-PDAC model, this system may provide a useful platform for studying how mitochondrial acinar injury, epithelial plasticity, stromal remodeling, and inflammatory signaling cooperate in pancreatic carcinogenesis.

## Data Availability

The data supporting the findings of this study are included in the article and Supplementary Materials. Additional raw data and materials are available from the corresponding author upon reasonable request, subject to institutional regulations and applicable material transfer agreements.

## Supplementary Materials

The following supporting information can be downloaded at: https://www.mdpi.com/article/10.3390/mps9040116/s1, Figure S1: Strain-dependent 24 h survival after single-dose L-Lysine administration; Figure S2: Gross appearance of the pancreas after L-Lysine-induced pancreatitis; Figure S3: Ultrastructural mitochondrial alterations after L-Lysine administration; Figure S4: Mitochondrial alterations are prominent in the peripheral region of the pancreas after L-Lysine administration; Figure S5: Efficient tamoxifen-induced Cre-loxP recombination in adult pancreatic exocrine cells; Figure S6: Representative additional examples of multifocal pancreatic neoplastic lesions after L-Lysine-induced pancreatitis; Figure S7: Gross appearance of advanced pancreatic tumor after L-Lysine-induced pancreatitis; Table S1: Primer sequences; Table S2: Primary antibodies; Table S3: Key Reagents and Materials.

## Abbreviations

The following abbreviations are used in this manuscript:

TAM	Tamoxifen
L-Lys	L-lysine
PDAC	Pancreatic ductal adenocarcinoma
ADM	acinar-to-ductal metaplasia
PanIN	pancreatic intraepithelial neoplasia
