# Supplementary material for: Single-Dose L-Lysine-Induced Pancreatitis Followed by Pancreatic Neoplastic Progression in Adult Kras/Trp53-Driven Mice
Source: Methods Protoc. 2026 Aug 10;9(4):116. doi: 10.3390/mps9040116 (PMC13516760; doi:10.3390/mps9040116)
Supplement: Supplementary file 1 [file mps-09-00116-s001.zip › mps-4447326-Supplementary Tables.pdf]

Supplementary Table S1. Primer sequences

Primer name	sequence
Ptf1a-CreER_F	CGTACTGACGGTGGGAGAAT
Ptf1a-CreER_R	CCCGGCAAAACAGGTAGTTA
Kras-G12D_wt_F	TGTCTTTCCCCAGCACAG T
Kras-G12D_mu_F	GCAGGTCGAGGGACCTAATA
Kras-G12D_R	CTGCATAGTACGCTATACCCTGT
p53-F	AGCTAGCCACCATGGCTTGAGTAAGTCTGCA
p53-R	CTTGGAGACATAGCCACACTG
Rosa26_F	AAAGTCGCTCTGAGTTGTTAT
Rosa26_M	GCGAAGAGTTTGTCCTCAACC
Rosa26_R	GGAGCGGGAGAAATGGATATG
RFP_F	GCTTCTTGTAGTCGGGGATG
RFP_R	TAAGCCTGCCCAGAAGACTCC

Supplementary Table S2. Primary antibodies

Target	Host species	Dilution	Supplier	Catalog no.	Main purpose
RFP	Rabbit	1:500	RCK	600-401-379	Lineage tracing
CK19	Goat	1:100	Santa Cruz	sc-33111	Ductal/PanIN marker
Pan-cytokeratin AE1/AE3	Mouse	1:100	DAKO	M3515	Epithelial marker
Amylase	Rabbit	1:1000	Sigma-Aldrich	A8273	Acinar marker
E-cadherin	Rabbit	1:100	Cell Signaling Technology	3195	Epithelial adhesion
β-catenin	Mouse	1:300	BD Biosciences	BD610153	Cell membrane marker
p-p44/42 MAPK	Rabbit	1:100	Cell Signaling Technology	4370	MAPK activation
TOM20	Rabbit	1:500	SantaCruz	sc-11415	Mitochondrial morphology

Supplementary Table S3. Key Reagents and Materials

Reagent/material	Supplier	Catalog no.	Use
Tamoxifen	Sigma-Aldrich	T5648	CreERT2 induction
Corn oil	Wako	032-07016	Tamoxifen solvent
L-lysine	Tokyo Chemical Industry	L0129	Pancreatitis induction
Physiological saline	Otuka	4987-035-08151-7	Vehicle control
4% paraformaldehyde	nacalai	09154-14	Tissue fixation
Alcian Blue solution, pH 2.5	Muto Pure Chemicals	Jan-85	Mucin staining
Contrast Red Solution	KPL	71-00-05	Counterstaining
Hematoxylin	Wako	131-09665	H&E/IHC counterstain
Eosin	Wako	054-06505	H&E staining
DAPI	ThermoFisher	D3571	Nuclear counterstain
DAB substrate	Vector Laboratories	94010	IHC detection
ImmPRESS HRP anti-rabbit IgG	Vector Laboratories	MP-7401	IHC secondary
ImmPRESS HRP anti-goat IgG	Vector Laboratories	MP-7405	IHC secondary
ImmPRESS HRP anti-mouse IgG	Vector Laboratories	MP-7422	IHC secondary
BZ-X800 microscope	Keyence		Bright-field/fluorescence imaging
LSM900 confocal microscope	Carl Zeiss		Confocal imaging
GraphPad Prism	GraphPad Software	Version 11.0.2	Statistical analysis
