# Supplementary material for: Single-Dose L-Lysine-Induced Pancreatitis Followed by Pancreatic Neoplastic Progression in Adult Kras/Trp53-Driven Mice
Source: Methods Protoc. 2026 Aug 10;9(4):116. doi: 10.3390/mps9040116 (PMC13516760; doi:10.3390/mps9040116)
Supplement: Supplementary file 1 [file mps-09-00116-s001.zip › mps-4447326-supplementary.pdf]

Supplementary Figures

Single-Dose L-Lysine–Induced Pancreatitis Followed by Pancreatic Neoplastic Progression in Adult *Kras*/*Trp53*-Driven Mice

Akihiro Kaneda, Masahiro Yoshida, Yoshiya Kawaguchi, and Kenichiro Furuyama *

Center for iPS Cell Research and Application, Kyoto University, Kyoto

A C57BL/6NCrSlc 6 week-old males

L-Lys dose	Saline	2.0	2.5	3.0 g/kg
24-h survival	3/3	4/4	3/4	0/3

B BALB/cCrSlc 6 week-old males

L-Lys dose	Saline	2.0	2.5	3.0 g/kg
24-h survival	3/3	4/4	4/4	2/4

Supplementary Figure S1. Strain-dependent 24 h survival after single-dose L-Lysine administration.
Six-week-old male C57BL/6NCrSlc and BALB/cCrSlc mice received a single intraperitoneal injection of physiological saline or L-Lysine at 2.0, 2.5, or 3.0 g/kg. Twenty-four-hour survival was assessed after injection. (A) In C57BL/6NCrSlc mice, all animals survived after saline or 2.0 g/kg L-Lysine administration, whereas survival decreased to 3/4 at 2.5 g/kg and 0/3 at 3.0 g/kg. (B) In BALB/cCrSlc mice, all animals survived after saline, 2.0 g/kg, or 2.5 g/kg L-Lysine administration, whereas 2/4 animals survived after 3.0 g/kg. These findings indicate that 2.0 g/kg was tolerated for 24 h in both genetically distinct mouse strains, whereas higher doses showed strain-dependent lethality.

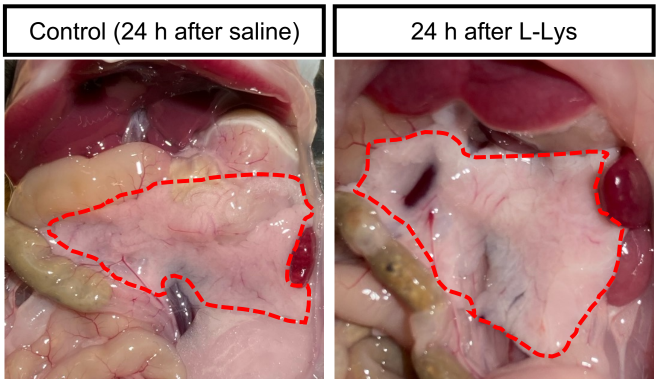

Supplementary Figure S2. Gross appearance of the pancreas after L-Lysine-induced pancreatitis. Representative macroscopic images of pancreata 24 h after physiological saline or 2.0 g/kg L-Lysine administration. The dashed red lines outline the pancreas. Compared with saline-treated controls, L-Lysine-treated pancreata showed gross edema-like changes consistent with acute pancreatitis.

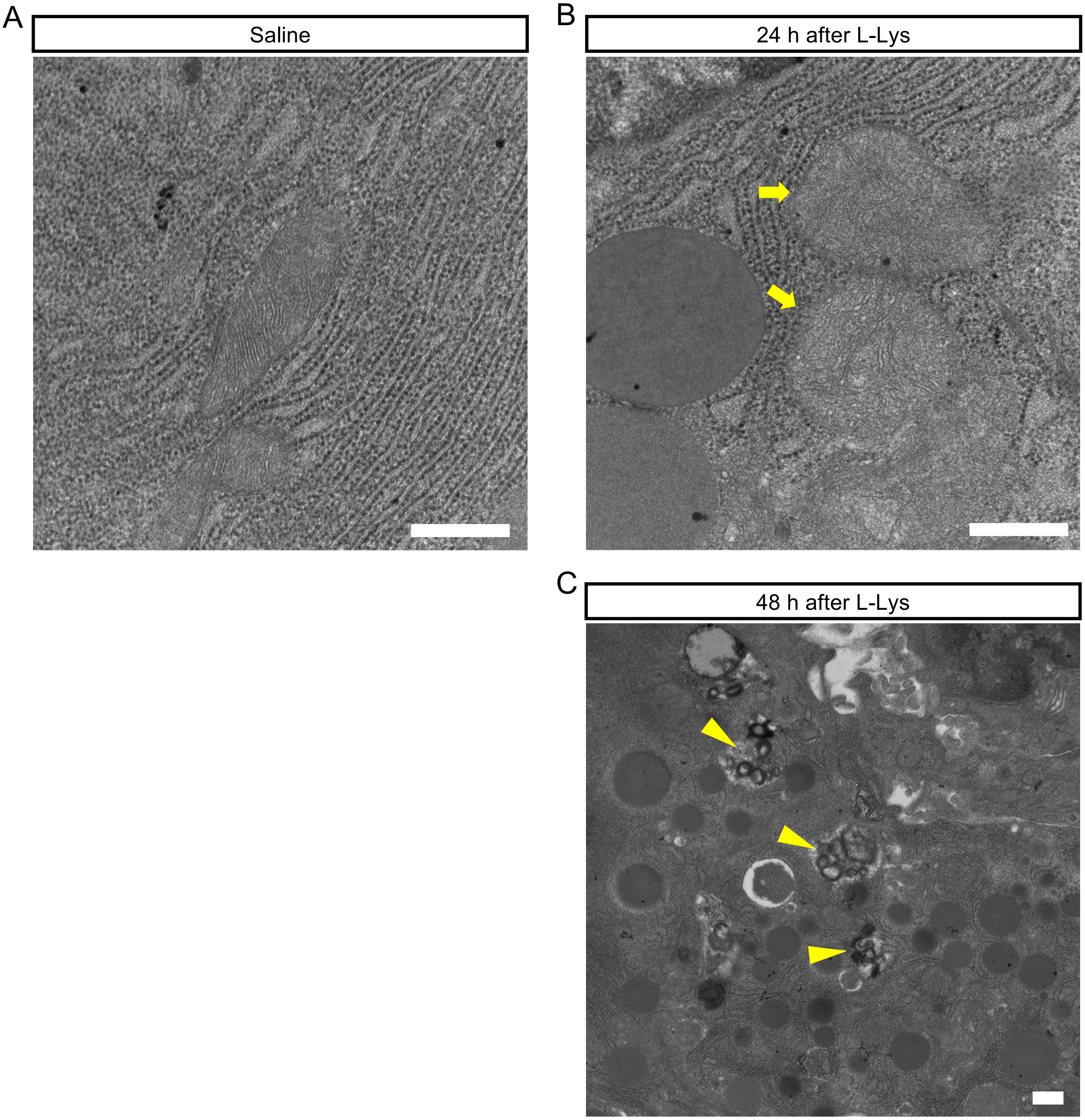

Supplementary Figure S3. Ultrastructural mitochondrial alterations after L-Lysine administration.

Representative transmission electron micrographs of pancreatic acinar cells from mice treated with physiological saline or L-Lysine. (A) Saline-treated control pancreas showing mitochondria with preserved oval morphology and densely packed, sharply defined, regularly aligned cristae. (B) At 24 h after L-Lysine administration, the indicated mitochondrion shows a swollen, rounded appearance, reduced matrix electron density, and rarefaction, fragmentation, and partial loss of cristae. (C) At 48 h after L-Lysine administration, double-membrane and multilamellar autophagic structures containing degenerating mitochondrial material are observed, consistent with mitophagosomes. Mitochondria remaining in the surrounding cytoplasm also show variable loss of cristae, reduced electron density, and vacuolar degeneration. Arrows indicate structurally altered mitochondria, and arrowheads indicate mitophagosome-like structures. Scale bars, 500 nm.

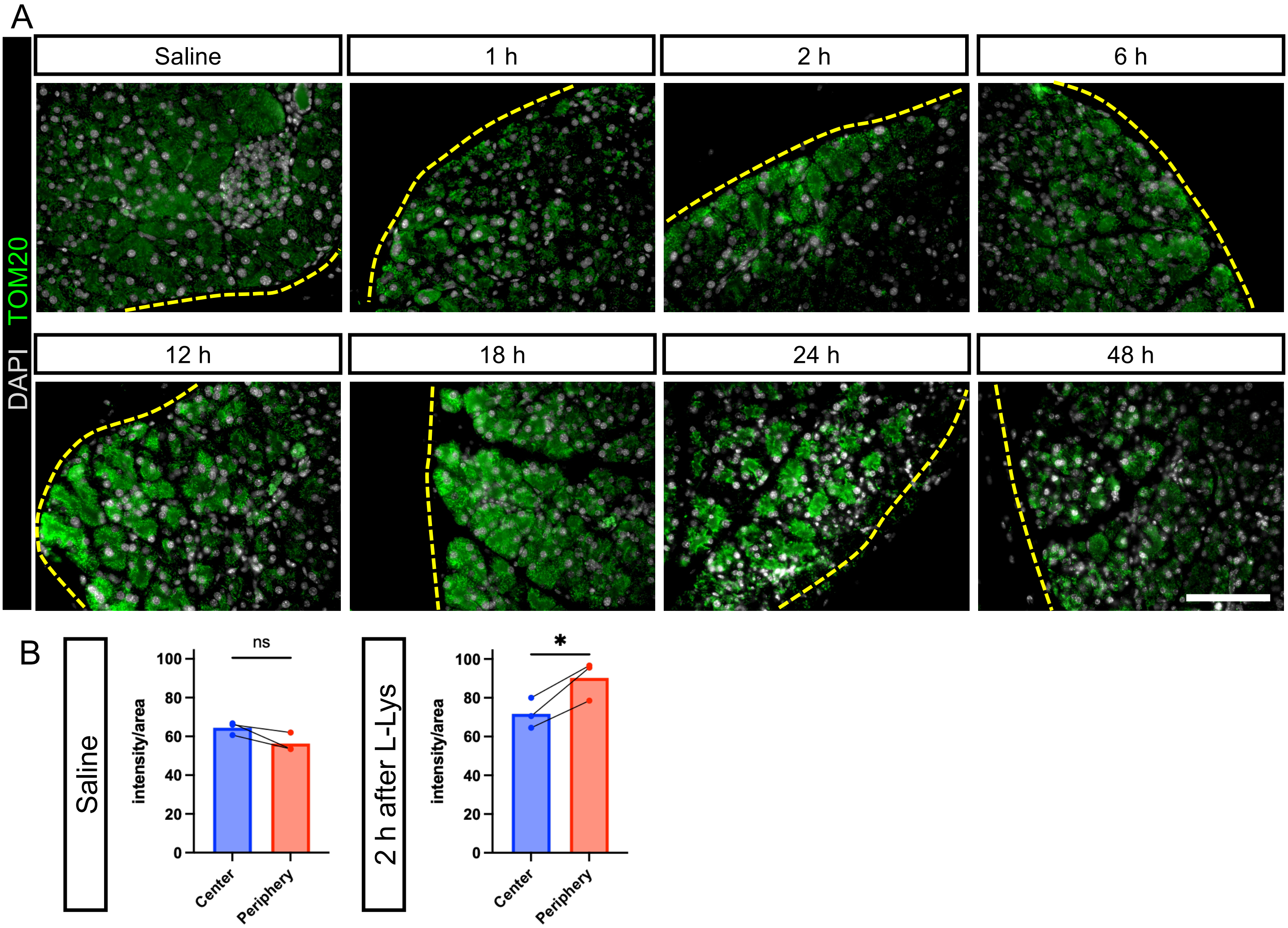

Supplementary Figure S4. Mitochondrial alterations are prominent in the peripheral region of the pancreas after L-Lysine administration.
(A) Representative immunofluorescence images of TOM20 and DAPI staining in pancreatic sections at the indicated time points after 2.0 g/kg L-Lysine administration. Saline-treated mice served as controls. The dashed yellow lines indicate the outer edge of the pancreas. TOM20 staining shows that mitochondrial alterations after L-Lysine administration are particularly evident in the peripheral pancreatic region. Scale bar, 100 μm.
(B) Mean TOM20 fluorescence intensity in the peripheral and central pancreatic regions of saline-treated mice and mice analyzed 2 h after L-Lysine administration. TOM20 fluorescence intensity was significantly higher in the peripheral region than in the central region at 2 h after L-Lysine administration, whereas no significant regional difference was detected in saline-treated mice. * $p < 0.05$; ns: not significant; two-tailed paired Student's t-test; $n = 3$ mice per group.

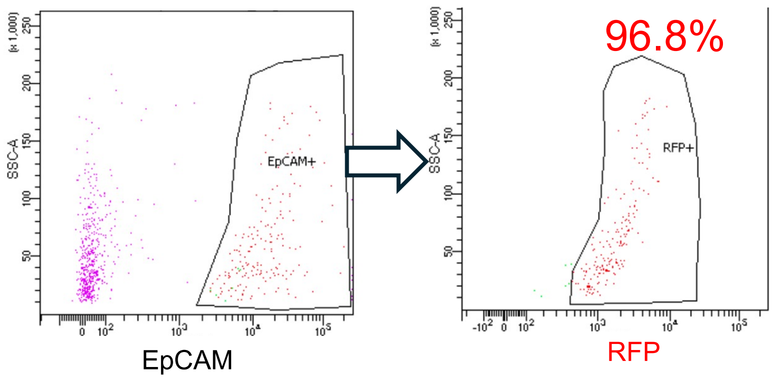

Supplementary Figure S5. Efficient tamoxifen-induced Cre-loxP recombination in adult pancreatic exocrine cells.

Three weeks after TAM administration, pancreatic tissues were dissociated into single-cell suspensions and stained with APC-conjugated anti-mouse EpCAM antibody and DAPI. Representative FACS plots show gating of live EpCAM-positive pancreatic exocrine cells and subsequent analysis of RFP expression. Among live EpCAM-positive cells, 96.8% were RFP-positive, confirming efficient TAM-induced Cre-loxP recombination in the adult *Ptf1a*-lineage pancreatic exocrine compartment.

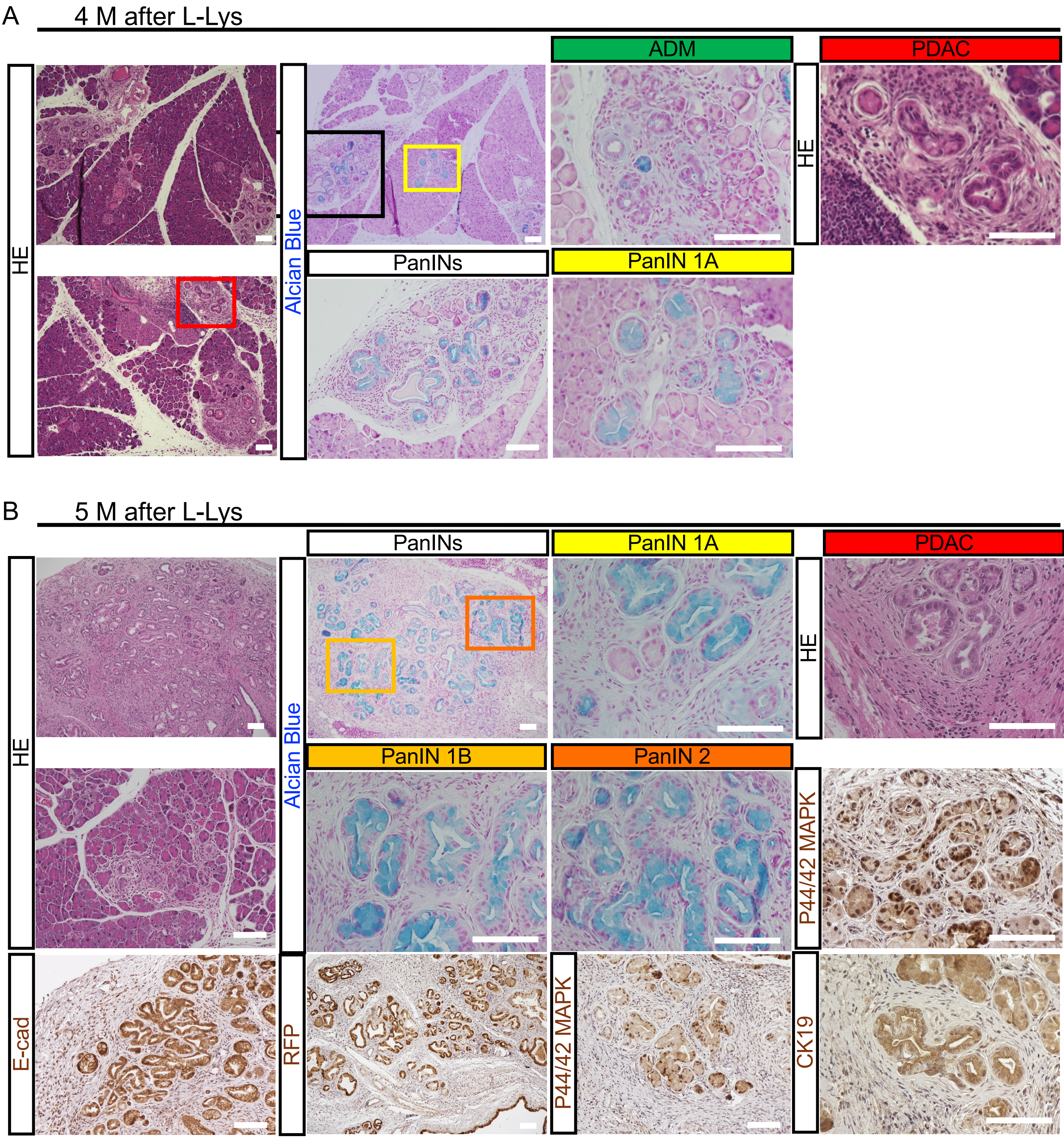

Supplementary Figure S6. Representative additional examples of multifocal pancreatic neoplastic lesions after L-Lysine-induced pancreatitis.
(A, B) Representative H&E, Alcian Blue, and immunohistochemical staining of pancreatic tissues from additional L-Lysine-treated *Ptf1a*^{CreER}; *Kras*$^{LSL\text{-}G12D}$; *Trp53*$^{LSL\text{-}R172H}$; Rosa26-RFP mice analyzed at 4 months (A) and 5 months (B) after L-Lysine administration. Similar to the 3-month sample shown in Figure 3, multifocal ADM, PanIN lesions, and PDAC were observed in independent mice. Alcian Blue staining highlights mucin-producing PanIN-like lesions. RFP immunostaining confirms the recombined *Ptf1a*-lineage origin of epithelial lesions. CK19 and E-cadherin staining demonstrate ductal epithelial differentiation, and phospho-p44/42 MAPK staining indicates MAPK pathway activation. Scale bars, 100 μm.

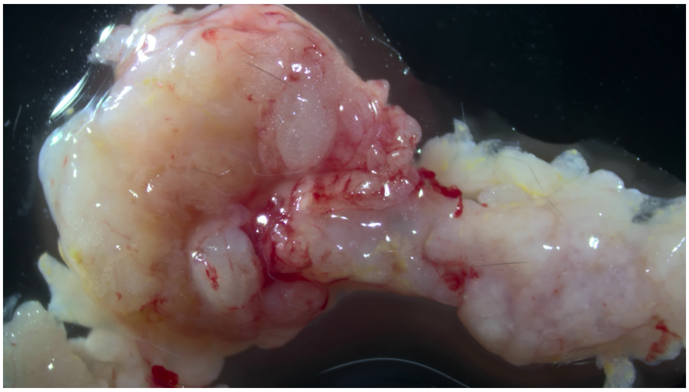

Supplementary Figure S7. Gross appearance of advanced pancreatic tumor after L-Lysine-induced pancreatitis.
Representative macroscopic image of the pancreas from an L-Lysine-treated *Ptf1a*^{CreER}; *Kras*$^{LSL\text{-}G12D}$; *Trp53*$^{LSL\text{-}R172H}$; Rosa26-RFP mouse at 5 months after L-Lysine administration. The image shows advanced pancreatic tumor formation following single-dose L-Lysine-induced pancreatitis.
